# {2-[(2-Carbamoylhydrazin-1-yl­idene)methyl-κ^2^ *N* ^1^,*O*]-5-meth­oxy­phenolato-κ*O* ^1^}chloridocopper(II)

**DOI:** 10.1107/S160053681203574X

**Published:** 2012-08-23

**Authors:** Roji J. Kunnath, M. R. Prathapachandra Kurup, Seik Weng Ng

**Affiliations:** a Department of Applied Chemistry, Cochin University of Science and Technology, Kochi 682 022, India; b Department of Chemistry, University of Malaya, 50603 Kuala Lumpur, Malaysia; c Chemistry Department, King Abdulaziz University, PO Box 80203 Jeddah, Saudi Arabia

## Abstract

The asymmetric unit of the title compound, [Cu(C_9_H_10_N_3_O_3_)Cl], contains two independent mol­ecules with similar structures. The Cu^II^ cation is *N*,*O*,*O′*-chelated by the deprotonated Schiff base ligand and is further coordinated by a Cl^−^ anion in a distorted ClNO_2_ square-planar geometry. In the crystal, adjacent mol­ecules are linked by N—H⋯O and N—H⋯Cl hydrogen bonds, forming a two-dimensional network parallel to [100].

## Related literature

For similar copper(II) complexes, see: Wang *et al.* (2008 ▶); Patole *et al.* (2001 ▶). 
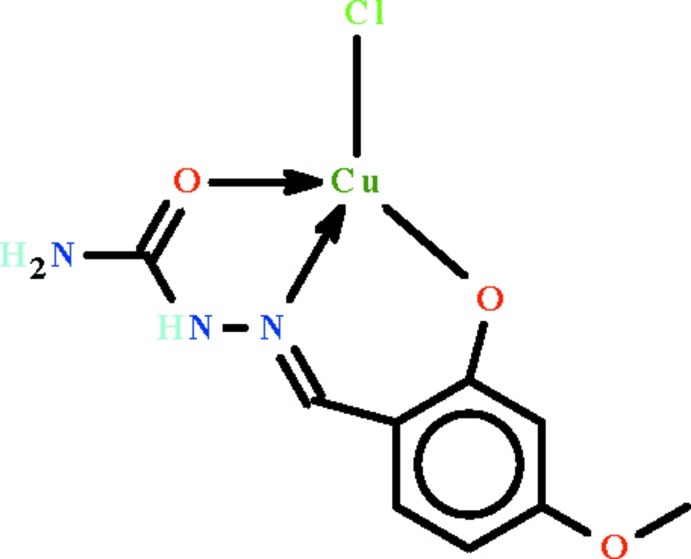


## Experimental

### 

#### Crystal data

[Cu(C_9_H_10_N_3_O_3_)Cl]
*M* *_r_* = 307.19
Monoclinic, 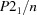

*a* = 11.8413 (6) Å
*b* = 14.2791 (7) Å
*c* = 13.5751 (6) Å
β = 102.501 (2)°
*V* = 2240.90 (19) Å^3^
*Z* = 8
Mo *K*α radiation
μ = 2.19 mm^−1^
*T* = 295 K
0.30 × 0.25 × 0.20 mm

#### Data collection

Bruker Kappa APEXII diffractometer
Absorption correction: multi-scan (*SADABS*; Sheldrick, 1996 ▶) *T*_min_ = 0.560, *T*_max_ = 0.669
20852 measured reflections
5562 independent reflections
3809 reflections with *I* > 2σ(*I*)
*R*_int_ = 0.047

#### Refinement

*R*[*F* ^2^ > 2σ(*F* ^2^)] = 0.040
*wR*(*F* ^2^) = 0.110
*S* = 1.02
5562 reflections
307 parameters
H-atom parameters constrained
Δρ_max_ = 0.57 e Å^−3^
Δρ_min_ = −0.37 e Å^−3^

### 

Data collection: *APEX2* (Bruker, 2010 ▶); cell refinement: *SAINT* (Bruker, 2010 ▶); data reduction: *SAINT*; program(s) used to solve structure: *SHELXS97* (Sheldrick, 2008 ▶); program(s) used to refine structure: *SHELXL97* (Sheldrick, 2008 ▶); molecular graphics: *X-SEED* (Barbour, 2001 ▶); software used to prepare material for publication: *publCIF* (Westrip, 2010 ▶).

## Supplementary Material

Crystal structure: contains datablock(s) global, I. DOI: 10.1107/S160053681203574X/xu5610sup1.cif

Structure factors: contains datablock(s) I. DOI: 10.1107/S160053681203574X/xu5610Isup2.hkl

Additional supplementary materials:  crystallographic information; 3D view; checkCIF report

## Figures and Tables

**Table 1:** Hydrogen-bond geometry (Å, °)

*D*—H⋯*A*	*D*—H	H⋯*A*	*D*⋯*A*	*D*—H⋯*A*
N2—H1⋯Cl2	0.88	2.42	3.203 (3)	149
N3—H2⋯O2^i^	0.88	2.01	2.824 (4)	153
N3—H3⋯Cl2	0.88	2.51	3.297 (3)	149
N5—H4⋯Cl1^ii^	0.88	2.40	3.212 (3)	154
N6—H5⋯O5^iii^	0.88	2.09	2.897 (3)	152
N6—H6⋯Cl1^ii^	0.88	2.56	3.351 (3)	149

Symmetry codes: (i) 
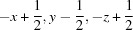
; (ii) 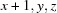; (iii) 
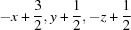
.

**Fig. 1.**
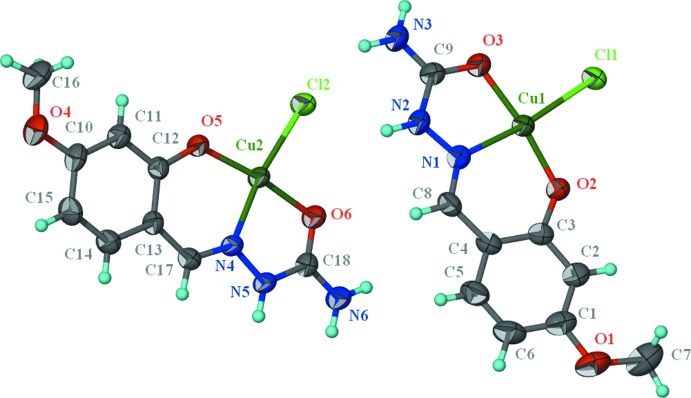
Thermal ellipsoid plot (Barbour, 2001) of CuCl(C9H10N3O3) at the 70% probability level; hydrogen atoms are drawn as spheres of arbitrary radius.

## supplementary crystallographic information

### Comment

Salicylaldehyde thiosemicarbazone and its subsituted derivatives are Schiff bases that are capable of *N*,*N*',*O*-chelation to transition metal ions, a feature that has been documented in a plethora of metal derivatives. Several copper derivatives have been reported (Wang *et al.*, 2008; Patole *et al.*, 2001). The Cu^II^ atom in CuCl(C_9_H_10_N_3_O_3_) (Scheme I) is *N*,*N*',*O*-chelated by the deprotonated Schiff base, and it exists in a square planar environment in the two independent molecules (Fig. 1). Adjacent molecules are linked by N–H···O and N–H···Cl hydrogen bonds to form a two-dimensional network (Table 1).

### Experimental

The Schiff base was prepared by heating 2-hydroxy-4-methoxybenzaldehyde (0.152 g,1 mmol) and semicarbazide hydrochloride (0.111 g,1 mmol) for 3 h. Copper(II) dichloride dihydrate (0.170 g, 1 mmol) was added to the solution and reflux was continued for another 2 h. Dark green colored crystals were isolated from the cool solution.

### Refinement

Carbon-bound H-atoms were placed in calculated positions (C–H 0.93 Å) and were included in the refinement in the riding model approximation, with *U*(H) set to 1.2*U*(C). The amino H-atoms were similarly treated (N–H 0.88 Å).

### Crystal data

**Table:**

[Cu(C_9_H_10_N_3_O_3_)Cl]	*F*(000) = 1240
*M**_r_* = 307.19	*D*_x_ = 1.821 Mg m^−^^3^
Monoclinic, *P*2_1_/*n*	Mo *K*α radiation, λ = 0.71073 Å
Hall symbol: -P 2yn	Cell parameters from 5495 reflections
*a* = 11.8413 (6) Å	θ = 2.3–27.0°
*b* = 14.2791 (7) Å	µ = 2.19 mm^−^^1^
*c* = 13.5751 (6) Å	*T* = 295 K
β = 102.501 (2)°	Prism, dark green
*V* = 2240.90 (19) Å^3^	0.30 × 0.25 × 0.20 mm
*Z* = 8	

### Data collection

**Table:**

Bruker Kappa APEXII diffractometer	5562 independent reflections
Radiation source: fine-focus sealed tube	3809 reflections with *I* > 2σ(*I*)
Graphite monochromator	*R*_int_ = 0.047
ω scans	θ_max_ = 28.4°, θ_min_ = 2.1°
Absorption correction: multi-scan (*SADABS*; Sheldrick, 1996)	*h* = −15→15
*T*_min_ = 0.560, *T*_max_ = 0.669	*k* = −17→19
20852 measured reflections	*l* = −11→18

### Refinement

**Table:**

Refinement on *F*^2^	Primary atom site location: structure-invariant direct methods
Least-squares matrix: full	Secondary atom site location: difference Fourier map
*R*[*F*^2^ > 2σ(*F*^2^)] = 0.040	Hydrogen site location: inferred from neighbouring sites
*wR*(*F*^2^) = 0.110	H-atom parameters constrained
*S* = 1.02	*w* = 1/[σ^2^(*F*_o_^2^) + (0.0443*P*)^2^ + 2.0625*P*] where *P* = (*F*_o_^2^ + 2*F*_c_^2^)/3
5562 reflections	(Δ/σ)_max_ = 0.001
307 parameters	Δρ_max_ = 0.57 e Å^−^^3^
0 restraints	Δρ_min_ = −0.37 e Å^−^^3^

### Fractional atomic coordinates and isotropic or equivalent isotropic displacement parameters (Å^2^)

**Table:**

	*x*	*y*	*z*	*U*_iso_*/*U*_eq_	
Cu1	0.29935 (3)	0.85386 (3)	0.32124 (3)	0.03357 (12)	
Cu2	0.79752 (3)	0.56618 (3)	0.33325 (3)	0.03219 (12)	
Cl1	0.11883 (7)	0.88631 (6)	0.33195 (7)	0.0418 (2)	
Cl2	0.61629 (7)	0.53124 (6)	0.33804 (8)	0.0509 (3)	
O1	0.6111 (2)	1.23052 (19)	0.4410 (2)	0.0569 (7)	
O2	0.35126 (19)	0.97847 (15)	0.33806 (18)	0.0365 (5)	
O3	0.25915 (19)	0.72346 (16)	0.2833 (2)	0.0446 (6)	
O4	1.1173 (2)	0.19137 (16)	0.4510 (2)	0.0488 (6)	
O5	0.85178 (17)	0.44138 (14)	0.35581 (17)	0.0334 (5)	
O6	0.75675 (19)	0.69642 (15)	0.29078 (18)	0.0380 (5)	
N1	0.4534 (2)	0.80675 (18)	0.3315 (2)	0.0324 (6)	
N2	0.4528 (2)	0.71236 (19)	0.3109 (2)	0.0396 (7)	
H1	0.5168	0.6802	0.3132	0.048*	
N3	0.3391 (3)	0.58301 (19)	0.2679 (3)	0.0495 (8)	
H2	0.2709	0.5559	0.2522	0.059*	
H3	0.4023	0.5497	0.2710	0.059*	
N4	0.9510 (2)	0.61509 (17)	0.34674 (18)	0.0276 (5)	
N5	0.9498 (2)	0.70935 (17)	0.3251 (2)	0.0330 (6)	
H4	1.0136	0.7425	0.3314	0.040*	
N6	0.8360 (2)	0.83611 (18)	0.2675 (2)	0.0400 (7)	
H5	0.7678	0.8625	0.2471	0.048*	
H6	0.8993	0.8692	0.2706	0.048*	
C1	0.5869 (3)	1.1399 (2)	0.4154 (3)	0.0409 (8)	
C2	0.4767 (3)	1.1034 (2)	0.3879 (2)	0.0357 (7)	
H2A	0.4132	1.1426	0.3843	0.043*	
C3	0.4589 (3)	1.0082 (2)	0.3654 (2)	0.0310 (7)	
C4	0.5574 (3)	0.9494 (2)	0.3722 (2)	0.0325 (7)	
C5	0.6682 (3)	0.9899 (3)	0.3998 (3)	0.0414 (8)	
H5A	0.7329	0.9519	0.4040	0.050*	
C6	0.6834 (3)	1.0825 (3)	0.4205 (3)	0.0459 (9)	
H6A	0.7576	1.1075	0.4380	0.055*	
C7	0.5192 (4)	1.2946 (3)	0.4323 (3)	0.0632 (12)	
H7A	0.5493	1.3555	0.4535	0.095*	
H7B	0.4679	1.2746	0.4741	0.095*	
H7C	0.4776	1.2974	0.3633	0.095*	
C8	0.5499 (3)	0.8510 (2)	0.3526 (2)	0.0340 (7)	
H8	0.6179	0.8176	0.3552	0.041*	
C9	0.3466 (3)	0.6727 (2)	0.2867 (3)	0.0366 (7)	
C10	1.0904 (3)	0.2828 (2)	0.4312 (2)	0.0349 (7)	
C11	0.9796 (3)	0.3175 (2)	0.4037 (2)	0.0326 (7)	
H11	0.9171	0.2770	0.3988	0.039*	
C12	0.9595 (2)	0.4135 (2)	0.3829 (2)	0.0268 (6)	
C13	1.0564 (3)	0.4741 (2)	0.3919 (2)	0.0291 (6)	
C14	1.1674 (3)	0.4347 (2)	0.4214 (3)	0.0376 (8)	
H14	1.2313	0.4740	0.4279	0.045*	
C15	1.1859 (3)	0.3423 (2)	0.4406 (3)	0.0412 (8)	
H15	1.2607	0.3188	0.4598	0.049*	
C16	1.0267 (3)	0.1256 (2)	0.4431 (3)	0.0516 (10)	
H16A	1.0587	0.0643	0.4593	0.077*	
H16B	0.9811	0.1257	0.3754	0.077*	
H16C	0.9788	0.1421	0.4891	0.077*	
C17	1.0479 (3)	0.5715 (2)	0.3717 (2)	0.0311 (7)	
H17	1.1158	0.6056	0.3767	0.037*	
C18	0.8432 (3)	0.7473 (2)	0.2934 (2)	0.0322 (7)	

### Atomic displacement parameters (Å^2^)

**Table:**

	*U*^11^	*U*^22^	*U*^33^	*U*^12^	*U*^13^	*U*^23^
Cu1	0.0244 (2)	0.0258 (2)	0.0503 (3)	−0.00060 (15)	0.00748 (16)	−0.00107 (17)
Cu2	0.0229 (2)	0.0211 (2)	0.0503 (3)	0.00078 (14)	0.00288 (16)	0.00072 (16)
Cl1	0.0258 (4)	0.0332 (4)	0.0670 (6)	−0.0013 (3)	0.0116 (4)	−0.0007 (4)
Cl2	0.0247 (4)	0.0293 (4)	0.0961 (8)	−0.0006 (3)	0.0073 (4)	−0.0012 (4)
O1	0.0594 (18)	0.0400 (16)	0.0694 (19)	−0.0185 (13)	0.0099 (14)	−0.0094 (13)
O2	0.0285 (12)	0.0257 (12)	0.0548 (15)	−0.0006 (9)	0.0081 (10)	−0.0012 (10)
O3	0.0277 (12)	0.0296 (13)	0.0749 (18)	−0.0020 (10)	0.0079 (11)	−0.0087 (12)
O4	0.0478 (16)	0.0282 (13)	0.0696 (18)	0.0131 (11)	0.0107 (12)	0.0097 (12)
O5	0.0226 (11)	0.0229 (11)	0.0524 (14)	0.0008 (8)	0.0031 (9)	−0.0013 (9)
O6	0.0301 (12)	0.0244 (12)	0.0585 (15)	0.0019 (9)	0.0074 (10)	0.0048 (10)
N1	0.0311 (14)	0.0290 (14)	0.0372 (15)	0.0004 (11)	0.0075 (11)	−0.0017 (11)
N2	0.0329 (15)	0.0260 (14)	0.0595 (19)	0.0039 (11)	0.0092 (13)	−0.0029 (13)
N3	0.0340 (17)	0.0280 (16)	0.085 (2)	0.0026 (12)	0.0091 (15)	−0.0099 (15)
N4	0.0266 (13)	0.0216 (13)	0.0342 (14)	−0.0005 (10)	0.0056 (10)	0.0025 (10)
N5	0.0277 (14)	0.0197 (13)	0.0507 (17)	0.0001 (10)	0.0068 (11)	0.0050 (11)
N6	0.0308 (15)	0.0249 (14)	0.0628 (19)	0.0023 (11)	0.0072 (13)	0.0098 (13)
C1	0.048 (2)	0.037 (2)	0.0372 (19)	−0.0124 (16)	0.0101 (15)	−0.0028 (15)
C2	0.0386 (19)	0.0313 (18)	0.0373 (18)	−0.0030 (14)	0.0085 (14)	0.0019 (14)
C3	0.0302 (17)	0.0312 (17)	0.0322 (17)	−0.0043 (13)	0.0081 (12)	0.0009 (13)
C4	0.0277 (16)	0.0359 (18)	0.0348 (17)	−0.0039 (13)	0.0086 (12)	0.0009 (13)
C5	0.0289 (18)	0.047 (2)	0.048 (2)	−0.0052 (15)	0.0064 (14)	−0.0006 (16)
C6	0.0335 (19)	0.052 (2)	0.050 (2)	−0.0151 (16)	0.0043 (15)	−0.0052 (17)
C7	0.086 (3)	0.041 (2)	0.060 (3)	−0.006 (2)	0.011 (2)	−0.0008 (19)
C8	0.0242 (16)	0.0371 (19)	0.0413 (19)	0.0014 (13)	0.0081 (13)	0.0018 (14)
C9	0.0308 (18)	0.0292 (17)	0.049 (2)	−0.0027 (13)	0.0069 (14)	−0.0014 (15)
C10	0.0394 (19)	0.0269 (17)	0.0378 (19)	0.0088 (13)	0.0071 (13)	0.0021 (13)
C11	0.0319 (17)	0.0275 (17)	0.0374 (18)	0.0013 (12)	0.0058 (13)	0.0003 (13)
C12	0.0248 (15)	0.0261 (16)	0.0287 (16)	0.0025 (11)	0.0042 (11)	−0.0012 (12)
C13	0.0258 (16)	0.0264 (16)	0.0346 (17)	0.0034 (12)	0.0052 (12)	−0.0008 (12)
C14	0.0257 (17)	0.0343 (19)	0.051 (2)	0.0021 (13)	0.0044 (14)	0.0026 (15)
C15	0.0286 (18)	0.038 (2)	0.056 (2)	0.0098 (14)	0.0055 (15)	0.0082 (16)
C16	0.060 (3)	0.0251 (18)	0.070 (3)	0.0024 (17)	0.015 (2)	0.0074 (17)
C17	0.0242 (16)	0.0297 (17)	0.0392 (18)	−0.0005 (12)	0.0063 (12)	−0.0002 (13)
C18	0.0305 (17)	0.0270 (16)	0.0397 (18)	0.0024 (13)	0.0087 (13)	−0.0001 (13)

### Geometric parameters (Å, º)

**Table:**

Cu1—O2	1.880 (2)	N6—H6	0.8800
Cu1—N1	1.920 (3)	C1—C2	1.380 (5)
Cu1—O3	1.963 (2)	C1—C6	1.396 (5)
Cu1—Cl1	2.2225 (9)	C2—C3	1.398 (4)
Cu2—O5	1.897 (2)	C2—H2A	0.9300
Cu2—N4	1.918 (2)	C3—C4	1.424 (4)
Cu2—O6	1.976 (2)	C4—C5	1.408 (4)
Cu2—Cl2	2.2179 (9)	C4—C8	1.429 (4)
O1—C1	1.354 (4)	C5—C6	1.356 (5)
O1—C7	1.408 (5)	C5—H5A	0.9300
O2—C3	1.319 (4)	C6—H6A	0.9300
O3—C9	1.257 (4)	C7—H7A	0.9600
O4—C10	1.357 (4)	C7—H7B	0.9600
O4—C16	1.412 (4)	C7—H7C	0.9600
O5—C12	1.311 (3)	C8—H8	0.9300
O6—C18	1.250 (4)	C10—C11	1.376 (4)
N1—C8	1.283 (4)	C10—C15	1.398 (5)
N1—N2	1.376 (4)	C11—C12	1.409 (4)
N2—C9	1.353 (4)	C11—H11	0.9300
N2—H1	0.8800	C12—C13	1.422 (4)
N3—C9	1.305 (4)	C13—C14	1.406 (4)
N3—H2	0.8800	C13—C17	1.417 (4)
N3—H3	0.8800	C14—C15	1.354 (4)
N4—C17	1.284 (4)	C14—H14	0.9300
N4—N5	1.377 (3)	C15—H15	0.9300
N5—C18	1.355 (4)	C16—H16A	0.9600
N5—H4	0.8800	C16—H16B	0.9600
N6—C18	1.314 (4)	C16—H16C	0.9600
N6—H5	0.8800	C17—H17	0.9300
			
O2—Cu1—N1	92.42 (10)	C6—C5—C4	122.0 (3)
O2—Cu1—O3	169.71 (10)	C6—C5—H5A	119.0
N1—Cu1—O3	81.91 (10)	C4—C5—H5A	119.0
O2—Cu1—Cl1	95.08 (7)	C5—C6—C1	119.5 (3)
N1—Cu1—Cl1	168.53 (8)	C5—C6—H6A	120.3
O3—Cu1—Cl1	91.89 (7)	C1—C6—H6A	120.3
O5—Cu2—N4	92.63 (10)	O1—C7—H7A	109.5
O5—Cu2—O6	169.86 (10)	O1—C7—H7B	109.5
N4—Cu2—O6	81.48 (10)	H7A—C7—H7B	109.5
O5—Cu2—Cl2	94.59 (7)	O1—C7—H7C	109.5
N4—Cu2—Cl2	169.05 (8)	H7A—C7—H7C	109.5
O6—Cu2—Cl2	92.49 (7)	H7B—C7—H7C	109.5
C1—O1—C7	118.8 (3)	N1—C8—C4	122.8 (3)
C3—O2—Cu1	127.6 (2)	N1—C8—H8	118.6
C9—O3—Cu1	112.7 (2)	C4—C8—H8	118.6
C10—O4—C16	118.8 (3)	O3—C9—N3	122.5 (3)
C12—O5—Cu2	127.32 (19)	O3—C9—N2	118.8 (3)
C18—O6—Cu2	113.1 (2)	N3—C9—N2	118.6 (3)
C8—N1—N2	119.5 (3)	O4—C10—C11	124.7 (3)
C8—N1—Cu1	129.0 (2)	O4—C10—C15	114.6 (3)
N2—N1—Cu1	111.46 (19)	C11—C10—C15	120.7 (3)
C9—N2—N1	115.0 (3)	C10—C11—C12	120.9 (3)
C9—N2—H1	122.5	C10—C11—H11	119.5
N1—N2—H1	122.5	C12—C11—H11	119.5
C9—N3—H2	120.0	O5—C12—C11	117.6 (3)
C9—N3—H3	120.0	O5—C12—C13	123.9 (3)
H2—N3—H3	120.0	C11—C12—C13	118.5 (3)
C17—N4—N5	119.8 (3)	C14—C13—C17	118.1 (3)
C17—N4—Cu2	128.5 (2)	C14—C13—C12	117.9 (3)
N5—N4—Cu2	111.69 (18)	C17—C13—C12	124.0 (3)
C18—N5—N4	115.1 (2)	C15—C14—C13	123.2 (3)
C18—N5—H4	122.4	C15—C14—H14	118.4
N4—N5—H4	122.4	C13—C14—H14	118.4
C18—N6—H5	120.0	C14—C15—C10	118.7 (3)
C18—N6—H6	120.0	C14—C15—H15	120.6
H5—N6—H6	120.0	C10—C15—H15	120.6
O1—C1—C2	124.5 (3)	O4—C16—H16A	109.5
O1—C1—C6	115.0 (3)	O4—C16—H16B	109.5
C2—C1—C6	120.5 (3)	H16A—C16—H16B	109.5
C1—C2—C3	121.0 (3)	O4—C16—H16C	109.5
C1—C2—H2A	119.5	H16A—C16—H16C	109.5
C3—C2—H2A	119.5	H16B—C16—H16C	109.5
O2—C3—C2	117.7 (3)	N4—C17—C13	123.3 (3)
O2—C3—C4	123.8 (3)	N4—C17—H17	118.3
C2—C3—C4	118.5 (3)	C13—C17—H17	118.3
C5—C4—C3	118.5 (3)	O6—C18—N6	123.2 (3)
C5—C4—C8	118.0 (3)	O6—C18—N5	118.5 (3)
C3—C4—C8	123.4 (3)	N6—C18—N5	118.2 (3)
			
N1—Cu1—O2—C3	−10.5 (3)	C2—C3—C4—C8	179.0 (3)
O3—Cu1—O2—C3	−66.8 (7)	C3—C4—C5—C6	0.5 (5)
Cl1—Cu1—O2—C3	160.8 (2)	C8—C4—C5—C6	−179.5 (3)
O2—Cu1—O3—C9	59.4 (7)	C4—C5—C6—C1	0.5 (5)
N1—Cu1—O3—C9	2.3 (2)	O1—C1—C6—C5	177.6 (3)
Cl1—Cu1—O3—C9	−168.0 (2)	C2—C1—C6—C5	−1.0 (5)
N4—Cu2—O5—C12	7.3 (3)	N2—N1—C8—C4	178.3 (3)
O6—Cu2—O5—C12	61.4 (6)	Cu1—N1—C8—C4	−0.6 (5)
Cl2—Cu2—O5—C12	−164.5 (2)	C5—C4—C8—N1	176.7 (3)
O5—Cu2—O6—C18	−55.8 (6)	C3—C4—C8—N1	−3.2 (5)
N4—Cu2—O6—C18	−0.9 (2)	Cu1—O3—C9—N3	177.4 (3)
Cl2—Cu2—O6—C18	169.9 (2)	Cu1—O3—C9—N2	−2.5 (4)
O2—Cu1—N1—C8	5.9 (3)	N1—N2—C9—O3	1.1 (5)
O3—Cu1—N1—C8	177.3 (3)	N1—N2—C9—N3	−178.8 (3)
Cl1—Cu1—N1—C8	−124.9 (4)	C16—O4—C10—C11	0.3 (5)
O2—Cu1—N1—N2	−173.0 (2)	C16—O4—C10—C15	−179.7 (3)
O3—Cu1—N1—N2	−1.7 (2)	O4—C10—C11—C12	−179.0 (3)
Cl1—Cu1—N1—N2	56.1 (5)	C15—C10—C11—C12	1.0 (5)
C8—N1—N2—C9	−178.2 (3)	Cu2—O5—C12—C11	174.7 (2)
Cu1—N1—N2—C9	0.9 (3)	Cu2—O5—C12—C13	−5.6 (4)
O5—Cu2—N4—C17	−5.5 (3)	C10—C11—C12—O5	179.1 (3)
O6—Cu2—N4—C17	−177.2 (3)	C10—C11—C12—C13	−0.7 (4)
Cl2—Cu2—N4—C17	125.8 (4)	O5—C12—C13—C14	−179.8 (3)
O5—Cu2—N4—N5	174.17 (19)	C11—C12—C13—C14	−0.1 (4)
O6—Cu2—N4—N5	2.46 (19)	O5—C12—C13—C17	−0.7 (5)
Cl2—Cu2—N4—N5	−54.6 (5)	C11—C12—C13—C17	179.0 (3)
C17—N4—N5—C18	175.9 (3)	C17—C13—C14—C15	−178.6 (3)
Cu2—N4—N5—C18	−3.7 (3)	C12—C13—C14—C15	0.5 (5)
C7—O1—C1—C2	−5.1 (5)	C13—C14—C15—C10	−0.2 (5)
C7—O1—C1—C6	176.3 (3)	O4—C10—C15—C14	179.4 (3)
O1—C1—C2—C3	−178.0 (3)	C11—C10—C15—C14	−0.6 (5)
C6—C1—C2—C3	0.5 (5)	N5—N4—C17—C13	−177.9 (3)
Cu1—O2—C3—C2	−170.5 (2)	Cu2—N4—C17—C13	1.7 (5)
Cu1—O2—C3—C4	10.1 (4)	C14—C13—C17—N4	−178.2 (3)
C1—C2—C3—O2	−178.9 (3)	C12—C13—C17—N4	2.7 (5)
C1—C2—C3—C4	0.5 (5)	Cu2—O6—C18—N6	179.5 (3)
O2—C3—C4—C5	178.4 (3)	Cu2—O6—C18—N5	−1.0 (4)
C2—C3—C4—C5	−1.0 (4)	N4—N5—C18—O6	3.2 (4)
O2—C3—C4—C8	−1.6 (5)	N4—N5—C18—N6	−177.2 (3)

### Hydrogen-bond geometry (Å, º)

**Table:**

*D*—H···*A*	*D*—H	H···*A*	*D*···*A*	*D*—H···*A*
N2—H1···Cl2	0.88	2.42	3.203 (3)	149
N3—H2···O2^i^	0.88	2.01	2.824 (4)	153
N3—H3···Cl2	0.88	2.51	3.297 (3)	149
N5—H4···Cl1^ii^	0.88	2.40	3.212 (3)	154
N6—H5···O5^iii^	0.88	2.09	2.897 (3)	152
N6—H6···Cl1^ii^	0.88	2.56	3.351 (3)	149

Symmetry codes: (i) −*x*+1/2, *y*−1/2, −*z*+1/2; (ii) *x*+1, *y*, *z*; (iii) −*x*+3/2, *y*+1/2, −*z*+1/2.
